# Supplementary material for: Are carbon emissions trading and green financial instruments synergistic? -Comprehensive quantitative research based on content analysis
Source: PLoS One. 2024 Mar 7;19(3):e0298601. doi: 10.1371/journal.pone.0298601 (PMC10919601; doi:10.1371/journal.pone.0298601)
Supplement: S1 Data — (ZIP) [file pone.0298601.s001.zip › Data/地级市Z_F工作报告.zip!/╔╜╬≈/╦╖╓▌╩╨/└┤╫╘╣1⁄2╓┌║┼í╛┬φ┐╦╩2╛▌═°í┐_2010.docx]

各位代表：

　　现在，我代表市人民政府向大会作工作报告，请予审议，并请市政协委员和其他列席人员提出意见。

　　一、2009年政府工作回顾

　　2009年是我们经受严峻考验、发展极为艰难的一年，也是全市上下攻坚克难、砥砺奋进、经济社会发展取得显著成就的一年。一年来，面对国际金融危机的严重冲击和经济结构深层次矛盾的重重困扰，市政府在省委、省政府和市委的坚强领导下，团结带领全市人民，坚持以邓小平理论和“三个代表”重要思想为指导，深入贯彻落实科学发展观，坚定信心，奋发向前，化危为机，全市经济社会在逆境中实现了平稳、协调、较快发展，全省瞩目，全国关注；各项重大工作走在全省前列，为长远发展打下坚实基础。

　　主要经济指标均保持较快增长，综合经济实力进一步增强。去年，我市经济发展在全省一路领先，在省政府考核的九项主要经济指标中，我市地区生产总值、工业增加值、社会消费品零售总额、海关进出口总额和城镇居民人均可支配收入五项指标增幅全省第一，一般预算收入和农民人均纯收入两项指标增幅全省第二。其中，地区生产总值完成561.3亿元，比上年增长10.6%;工业增加值完成270.2亿元,增长18%;财政总收入达到110亿元,增长7.8%；一般预算收入达到46.2亿元（含煤矿兼并重组一次性资源价款），增长22.3%；全社会固定资产投资完成301.5亿元，增长35.9%；社会消费品零售总额达到128.6亿元，增长23.1%；海关进出口总额达到1.13亿美元，增长86.5%；城镇居民人均可支配收入达到15500元，增长10.7%；农民人均纯收入达到5124元，增长8.3%。

　　重点工程建设规模空前，发展后劲大为增强。紧紧抓住中央扩内需的重大战略机遇，启动实施了以基础设施、民生工程和产业结构调整项目为主的162项重点工程，总投资1304亿元，项目之多，规模之大，前所未有。当年完成投资379.9亿元，成为拉动经济增长的重要引擎。图书馆、博物馆、体育馆、文化艺术中心等60多项工程竣工使用，城市功能大为完善，城市面貌发生了巨大变化。特别是引黄北干、平朔东露天煤矿、同煤麻家梁煤矿、准朔铁路、山阴至灵丘高速公路、大西客运线等国家级重点工程的开工建设，将为朔州的长远发展提供强有力支撑。

　　产业结构调整取得重大突破，转型发展迈出较大步伐。制定实施了九大产业调整振兴规划，传统产业快速优化升级，新兴产业加速发育成长。煤矿兼并重组取得重大成果，产业素质得到根本性提高。全市135座地方煤矿重组整合为67座，年产30万吨以下矿井全部淘汰，矿井数量减少一半；平均单井生产规模将由45万吨提高到131万吨，增加两倍；产能将由6156万吨提高到8780万吨，增长43%；资源回收率将由20%左右提高到75%以上。所有保留矿井将全部实现机械化综采，全部达到90万吨以上规模，煤炭工业的集约化、规模化、机械化水平和资源回收率、安全保障能力都将有一个大跨越，全市煤炭工业进入全新的发展阶段。电力工业快速壮大。大力发展煤矸石电厂、热电联供电厂和风力发电项目，新增电力装机容量70万千瓦，开工建设230万千瓦，新型能源工业基地地位得到进一步巩固。新兴产业掀起发展高潮。有 30多个投资亿元以上的非煤非电工业项目开工建设，新型材料、陶瓷、制药等产业快速壮大。特别是建材业发展迅猛，全市将形成10亿块空心蒸压砖、50万立方粉煤灰砌块和1300万吨水泥的生产能力，煤—电—新型建材循环经济链条基本形成。服务业加速发展。总投资达到110亿元，占到全市固定资产投资总额的36.5％。应县木塔和右玉县成为国家4Ａ级旅游景区，旅游业发展日益兴旺。招商引资继续保持良好势头。全市140个招商项目共到位外来投资60多亿元，为产业结构调整和经济发展增添了活力。

　　生态文明建设取得新成效，宜居城市建设迈出新步伐。坚决淘汰落后产能，大力关闭污染企业，积极推广节能工程，万元地区生产总值综合能耗下降5.97％，提前一年完成“十一五”二氧化硫和化学需氧量减排任务。市区二级以上天数达到340天，首次达到国家二级以上标准。低碳经济发展模式引起全国全省关注，被评为“中国十佳低碳贡献城市”，神头一电厂荣获“中国节能减排二十佳企业”称号。坚持生态优先原则，完成造林33万亩，被评为“全省造林绿化先进市”和“全省林业建设先进市”。

　　实施更加有力的强农惠农政策，“三农”工作进一步加强。在认真落实国家和省各类补贴政策的基础上，进一步加大对玉米、甜菜、小杂粮、大棚种植和养殖小区的补贴力度，并扶持种粮大户发展规模经营。市县财政贴息，支持金融部门向农民发放了6600万元大棚贷款和1亿元奶牛养殖贷款。全市共落实各级各类惠农补贴2.6亿元，农民人均增收232元。在各项强农惠农政策的有力支持下，农业生产战胜了五十年一遇的严重干旱，实现了稳定发展。粮食产量达到7.2亿公斤，仍是历史上第四个高产年。奶牛养殖业实现平稳回升，走上规模化、标准化健康养殖的发展轨道，入园奶牛占到奶牛总数40%。农民人均纯收入超过全省平均水平880元，被省委评为“增加农民收入先进市”。大力实施农村“五个全覆盖工程”，新农村建设取得明显成效。投资8.36亿元，新修通村水泥路1225公里，覆盖113个村，93.9％的建制村通了水泥（油）路；兴建165处饮水工程，使13万农民受益；加固改造校舍27.2万平方米，133所农村中小学办学条件得到改善；新建682个农村卫生室，并为所有农村卫生室配齐村医，提前一年完成全覆盖任务，全省领先；1010个村通达有线电视，426个村实现了卫星覆盖，占到建制村总数的85.3%。新增新农村建设重点推进村120个，新农村试点村达到420个，占到全市农村总数的四分之一。扶贫工作取得新进展，8550名农村贫困人口实现脱贫。

　　以改善民生为重点的各项社会事业加快发展，和谐社会建设卓有成效。坚持越是困难时期越要更加注重改善民生，大幅度增加了社会事业投入，社会事业发展滞后的状况正在加快改变。教育事业均衡发展。农村寄宿制学校建设成效突出，荣获全国“义务教育均衡发展先进地区”称号。高等教育加速发展。师专建设进展顺利，山西电力专科学校朔州分校（能源学院）奠基。医疗卫生事业进一步发展。县乡村三级医院大部分进行了改扩建，新型农村合作医疗参合率和补偿水平大幅度提高。甲型H1N1流感疫情得到有效防控。人口和计划生育工作获得省政府目标责任制考核综合先进奖。科技创新能力进一步提高，全年申请专利153件，提前完成“十一五”计划任务。就业再就业工作扎实有效，新增城镇就业岗位1.86万个，超省定目标21%。社会保障体系进一步完善，各项社保基金积累达到17.5亿元，比上年增长40%；农村五保户供养水平、城乡低保对象保障水平、企业退休人员基本养老金都有新的提高。大力推进保障性安居工程，开工建设各类保障性住房4.51万套、363.6万平方米，完成投资53.77亿元。住房公积金覆盖面进一步扩大，综合业务考评全省第一。支援四川地震灾区重建工作圆满完成任务。市县两级财政补贴1亿多元，保证了冬季供暖平稳进行。认真开展了安全生产专项整治，健全了安全生产管理机制，特别强化了煤炭行业安全生产长效机制，事故起数和死亡人数均大幅度下降，被评为全省“安全生产专项整治先进市”和“安全生产工作优秀市”。认真做好信访工作，全面加强社会治安综合治理，扎实做好国庆六十周年安保工作，社会秩序和谐稳定，被省委、省政府评为“和谐社会建设先进市”。积极支持国防后备力量建设，人防工作取得突破，双拥工作深入开展。民族宗教、档案史志、地震、气象、老龄、残疾人等各项社会事业都取得了新成绩。

　　民主法治建设稳步推进，精神文明建设成果丰硕。市政府自觉接受人大监督，积极支持市政协及各民主党派、人民团体和无党派人士参政议政，办理人大代表建议66件，政协委员提案165件。坚持依法行政，不断加强政府法制建设，全国首创“行政复议区域联办制度”，引起广泛关注。深入开展廉政建设，扎实推进煤焦领域反腐败斗争，累计清缴各类基金和资源价款18亿元。加强文化设施建设，推进文化体制改革，文化事业日益繁荣。扎实开展讲文明、树新风活动，被评为“山西省2008—2009年度文明和谐城市”。隆重庆祝建市二十周年，进一步激发了全市人民的自信心和自豪感。在艰苦奋斗中铸就的右玉精神，成为激励全省人民不断前进的精神风范。

　　各位代表，现在，我十分欣慰和自豪地郑重向大家报告：经过全市人民一年的艰苦努力，市五届人大一次会议所确定的2009年经济社会发展目标任务均已圆满或超额完成！而且还做了许多打基础、利长远的大事实事，为朔州未来腾飞奠定了更加坚实的基础！在增强硬实力的同时也赢得了诸多荣誉，新华社《国内动态清样》和《内部参考》连续六次介绍“朔州现象”、“朔州经验”，省委省政府肯定我们是全面发展、全面进步，并被评为2009中国十座“最具创新力城市”之一，进一步扩大了朔州的知名度，增强了朔州经济社会发展的软实力！

　　在去年那样极端困难的形势下，能够取得这样的成绩，实属不易。这是省委、省政府和市委正确领导的结果，是市人大、市政协有力监督和鼎力支持的结果，是全市人民团结奋斗、辛勤劳动的结果。在此，我代表市人民政府，向全市人民和所有关心支持朔州改革发展的各界人士，表示崇高的敬意和衷心的感谢！

　　回顾一年的实践，我们之所以能够取得这样的好成绩，主要有三个方面的原因：一是连续数年坚持不懈的科学发展为我们奠定了一个好基础。长时期以来，全市上下致力于科学发展，致力于化解影响和制约全市经济社会发展的深层次矛盾，及早预见到单一传统能源工业所潜在的危机，大力推进产业结构优化升级，特别是在传统产业新型化上下了很大功夫，煤矿机械化改造和兼并重组全省领先，成为支持经济发展的主动力。大力实施了输煤变输电战略，积极加快电力工业发展，特别是建设了一批煤矸石电厂和风力发电项目，确保了电力工业稳定发展。建设了一批非煤非电工业项目，大力发展了服务业，形成了一批新的经济增长点，为保增长作出了贡献。二是危机面前快速出击为我们赢得了好机遇。在危机初现之时，我们就及时采取了一系列措施，打出了一套组合拳，抢占了先机。特别是在争资金、争项目上，反应快，力度大，取得了项目总数和投资规模全省靠前、县均第一的好成绩，为拉动当前经济增长、奠定长远发展基础创造了条件。三是心齐气顺的干部队伍和踏实苦干的工作作风为我们营造了攻坚克难、干事创业的好氛围。面对危机带来的困难和压力，全市上下信心不减，精神不倒，不等待，不退缩，心往一处想，劲往一处使，齐心协力，共克时艰，越是困难越向前，越是重压越奋进，全市上下真正形成了同心同德、奋发向前的大好局面，这是我们成功的根本动力源泉！

　　实践证明，只要我们始终坚持以科学发展观为指导，按照转型发展、安全发展、全面发展、和谐发展的方向，紧紧抓住影响和制约全市经济社会发展的重大问题，坚定不移、坚持不懈地抓下去，就一定能够更加有效地增强全市经济社会发展的持续性、稳定性、更加有效地应对各种冲击，切实增强全市的综合经济实力和可持续发展能力，使全市经济社会发展更加健康、更加稳定！只要我们能够客观冷静地分析形势，正确把握机遇，真正做到困难面前不退缩、机遇面前不迟疑，敢出手、快出手，就一定能够达到有予则立，化危为机，赢得主动，赢得先机！只要我们能够始终保持蓬勃向上、同心同德、心齐气顺的良好氛围和不畏艰难、无私奉献、踏实苦干的良好作风，就一定能够战胜一切困难！万事皆在人为。我们有这样一批优秀的干部队伍，有全市155万敢于吃苦奉献的人民群众，一切艰难险阻都会在我们面前低头，胜利终将属于我们！

　　二、2010年政府工作总体要求和主要目标任务

　　今年是有效战胜国际金融危机、保持经济平稳较快发展的关键一年，也是全面实现“十一五”规划目标并为“十二五”发展打好基础的重要一年。做好今年的工作，意义重大。

　　今年的经济形势，总体上要好于去年。从外部大环境看，世界经济好转迹象明显，有望实现恢复性增长；我国经济回升向好势头得到巩固，扩内需政策效应继续显现。宏观经济形势的好转，必将对我市经济产生积极影响。从我市自身而言，随着煤矿兼并重组的到位和机械化改造的加快推进，重点工程作用的逐步显现，经济增长的内生力显著增强。但是，我们也要清醒地认识到，宏观经济回升向好的基础还不牢固，面临的形势还很复杂；特别是我市经济社会发展中还存在着不少突出问题和困难。中小企业经营困难，企业开工依然不足，市场需求还不稳定；产业结构调整任务艰巨而紧迫，低碳经济的兴起又给我们带来更高的门槛和制约，资源环境压力进一步加大；农业基础薄弱，农民持续增收难度较大；统筹城乡和经济社会发展任务繁重，教育、医疗、住房、社会管理等方面的突出问题亟待解决；政府职能转变、部分干部的工作作风与当前形势要求还有不小差距，等等。我们一定要全面正确地判断形势，既要看到困难和问题，增强忧患意识；更要充分利用有利条件和积极因素，努力化解矛盾，强化机遇意识和发展意识，坚定信心，扎实工作，推动科学发展，促进社会和谐。

　　今年政府工作的总体要求是：认真贯彻党的十七大和十七届三中、四中全会精神，以邓小平理论和“三个代表”重要思想为指导，深入贯彻落实科学发展观，紧紧围绕转型发展、安全发展、和谐发展、全面发展要求和建设最宜居最宜发展城市目标，着力抓好产业优化升级、基础设施建设、城乡一体化发展、生态文明建设、改善民生民计和民主法治建设六个重点，加快推进经济发展方式转变，提高发展质量和效益，切实提升全市综合经济实力、可持续发展能力、社会和谐水平和人民生活质量，在新一轮发展中实现率先科学发展！

　　今年经济社会发展的主要预期目标是：地区生产总值达到630亿元，增长10%；规模以上工业增加值达到310亿元，增长14%；全社会固定资产投资达到377亿元，增长25%；财政总收入达到121亿元，增长10%；一般预算收入达到44.8亿元，同口径增长10.7%；社会消费品零售总额达到150亿元，增长16.6%；居民消费价格涨幅控制在3%左右；城镇居民人均可支配收入达到17050元，增长10%；农民人均纯收入达到5630元，增长10%。

　　在完成上述指导性指标的同时，要确保完成以下约束性指标:万元地区生产总值综合能耗下降7.1%，二氧化硫、化学需氧量排放量分别控制在13.8万吨和1.15万吨以内，城镇登记失业率控制在4%以内，人口自然增长率控制在6‰以内。

　　要重点抓好六个方面的工作：

　　（一）着力构建低碳经济体系，打造新型绿色低碳经济基地

　　加快经济结构调整，推动产业优化升级，促进发展方式转变，是经济建设的永恒主题，对我市这样支柱产业单一的资源型地区而言，更是一项刻不容缓的重大战略任务。我市产业结构调整和优化升级的方向，唯一正确的选择就是发展低碳经济，着力打造新型绿色低碳经济基地。发展以低耗能、低排放、低污染为主要特征的低碳经济，是国际社会普遍倡导和推崇的经济发展模式，是经济发展到一定程度的必然要求，也是世界各国共同应对气候变暖而采取的共同行动。世界在关注着中国的低碳经济发展，党中央、国务院对低碳经济发展十分重视，省委省政府更明确提出，“朔州要为全省低碳经济发展闯出一条路子”。经济发展的迫切需要，党和政府的殷切期望，都要求我们必须牢固树立低碳发展、循环发展、绿色发展的理念，以九大产业调整振兴规划为指导，以构建低碳经济体系为核心，以传统产业升级改造、新兴产业发育壮大、大力发展循环经济、强力推进节能减排为抓手，着力推进单一产业结构向多元产业结构转变，低端产业向高端产业转变，高碳产业向低碳转型发展，粗放增长向绿色崛起迈进。

　　大力推进煤炭产业优化升级，强化高碳产业改造。巩固煤炭资源整合、煤矿兼并重组成果，按照有关法规政策，妥善处理好各项后续工作，善始善终地搞好煤矿兼并重组工作，提高煤炭产业集中度和集约化经营水平。加快煤矿复工复产进程，充分发挥煤炭产业支柱地位。突出抓好煤矿机械化改造和标准化矿井建设，积极推进煤炭洗选加工，提高煤炭工业机械化、信息化洁净化水平，提高资源回收率，减少资源浪费和环境污染。力争到明年年底，使全市所有保留矿井全部建成一级安全质量标准化矿井。同时，要按照“六条标准”，年内将68座淘汰矿井彻底关闭，严禁非法生产。严格实行公路煤炭销售统一管理，规范煤炭运销秩序。

　　积极培育新兴产业，加快低碳产业发展。加快发展电力工业。抓好已开工的四座煤矸石电厂和热电联供电厂、两座风力电场建设，积极推进神头一电厂关小上大2×60万千瓦项目早日开工，使全市电力装机容量尽快达到800万千瓦。特别要高度重视并积极开发利用风能、太阳能、生物质能等新型低碳能源和再生能源，制订完善风力电场建设规划，支持光伏发电、秸秆发电和垃圾焚烧发电项目建设。大力培育非煤电工业。煤化工业要巩固提升以合成氨、甲醇、多孔硝铵为主的传统化工，积极发展橡胶助剂、塑料助剂等精细化工。冶金业要以市场为导向，淘汰落后产品，开发新产品，实现产业振兴。机械制造业要以服务主导产业为重点，从设备维修和配件加工入手，向技术集成、设备成套方向发展，逐步壮大产业规模。新型材料业要依托资源优势，着力开发节能环保新材料和高技术产品。积极推进墙体材料革新，逐步限制粘土砖，推广粉煤灰制砖和矸石制砖，政府投资项目必须使用环保新材料。食品业要提高加工转化水平，做强酒类、乳业等传统优势产品，做精小杂粮、肉类、油类等特色产品，带动农业产业化发展。加快发展服务业。巩固服务业加速发展的良好势头，努力提升服务业发展水平及其在国民经济中的比重。统筹发展现代物流、机电维修、商贸流通、酒店餐饮、市政公用事业、社区物业管理等服务业，开工建设交通物流中心、供销社“三农”服务中心、煤炭物流交易中心。积极推进“三网”融合。突出抓好旅游和文化产业。加快编制旅游业发展总体规划，筹划建立旅游业发展基金。深入挖掘文化旅游资源，加强景点景区建设，着力打造特色旅游品牌，把应县木塔景区、右玉生态旅游区打造成一流景区，把山阴广武、怀仁金沙滩、朔城区神头海、平鲁乌龙洞打造成观光休闲游的新亮点。注重文化产业发展，加大文化产业投入，鼓励文化创作，努力推出一批地域特色鲜明、适合旅游观赏的演艺节目和文化产品，实现文化与旅游产业互动发展。

　　大力发展循环经济，提高资源利用率。要以园区为依托，以项目建设为突破口，积极推进循环经济发展，实现资源再利用，在循环利用中实现减碳。重点抓好已列入全省“十二五”工业整体规划的“三大工业基地”（乳品、电力、陶瓷）、“三大产业集群”（高岭土、特色食品、新型建材）和“九大工业园区”（市经济技术开发区、朔城区富甲工业园区、怀仁云东工业园区、平鲁循环经济园区、山阴金海洋循环经济园区、平朔循环工业园区、右玉威远工业园区、应县城东工业园区、神头循环经济园区），坚持基础设施共享、项目集中布局、产业联动发展、资源循环利用，借助煤炭资源优势，延长煤电冶、煤化工、煤建材等产业链条。突出抓好一批循环经济项目。抓紧启动以神头二电厂马邑灰库为依托的粉煤灰综合利用园区，下大力气抓好利用粉煤灰提取氧化铝和白炭黑项目。

　　强力推进节能减排，强化源头控制和末端治理。健全节能减排监察体系和评价考核体系，严格实行节能减排目标责任制。狠抓节能工程，从源头上控制碳产生。深入开展煤炭、电力、冶金、化工、建材等高耗能行业能效达标活动，加强对重点能耗企业的节能监管；积极推广节能技术，推动节能建筑示范工程建设，提倡全社会节能。继续实施“蓝天碧水工程”，全面推进资源环境保护工作，着力减少碳排放。加强燃煤电厂脱硫项目建设和运行监管。加大集中供热工程改造力度，新上以神头二电厂为热源的市区热电联供工程，彻底解决市区热源不足、供热布局分散、运行低效的问题。加快建设污水处理厂中水回用工程，加强企业废水达标治理、城镇污水处理和循环利用，加强饮用水源地保护。

　　同时，要千方百计促进中小企业发展。建立和完善中小企业服务体系，启动建设全市工业产品电子商务平台和工业产品展示中心。加大财政扶持力度，每年拿出一定专项资金，支持企业技术改造和新项目建设。继续实施企业社会保险“五缓四减三补”政策，减轻企业负担。加强对中小企业的金融支持。进一步壮大中小企业信用担保体系，积极发展小额贷款公司，鼓励、争取有关股份制商业银行设立分支机构，拓宽融资渠道。建立银企洽谈稳定机制，促进银企合作。鼓励有实力的骨干企业到资本市场直接融资。

　　各位代表，发展低碳经济，是社会进步的必然要求，是确保朔州持续健康发展的必然选择，既是一道重大经济课题，也是一项重大政治任务。我们一定要超前谋划，把握先机，率先突破，努力当好全省低碳经济发展闯路人与排头兵，为朔州未来发展闯出一条新路子，开辟一片新天地！

　　（二）坚持不懈推进重点工程建设，增强经济社会发展后劲

　　要牢牢抓住国家继续实施扩内需政策的机遇，坚持基础设施与产业开发并重、国有投资与民间投资并举，巩固发展重点工程建设的大好势头，再掀重点工程建设新高潮，充分发挥好投资拉动经济增长、推动社会发展的积极作用。

　　切实抓好一批关系全市未来发展的重大骨干项目。启动实施300项重点工程，其中续建项目144个，新开工项目156个，总投资1348亿元，年内力争完成投资400亿元。铁路公路方面，着力抓好准朔铁路、大西客运线、大呼高速公路、灵丘至山阴高速公路等续建项目，开工建设山阴至平鲁高速公路、朔州至平鲁高速公路、神电运煤通道二期工程、广武旅游公路和大忻线改造工程、虎山线改建工程，全面完成村通水泥路“全覆盖”任务，尽快实现“县县通高速、村村通油路”。水利设施方面，加快建设引黄北干线及大梁水库、耿庄水库、尚希庄水库、海子湾水库，加快推进桑干河上游生态环境综合治理工程。城市建设方面，继续抓好朔州古城改造、城中村改造、保障性住房和城市道路建设。产业结构调整方面，着力抓好平朔东露矿、同煤麻家梁矿井建设及同煤200万吨干法水泥等项目。同时在教育、医疗等社会事业方面推进一批重点项目建设，提高公共服务能力。

　　要努力创新投资机制。加快重点工程建设，投资是关键。要加强沟通协调，努力争取上级支持，并继续紧扣中央和省投资政策及方向，优化筛选一批新项目，争取列入上级投资规划，使更多项目和资金在我市落户。进一步加大招商引资力度，吸引更多外来资金。要以“中博会”、“能源博览会”等大型招商引资活动为平台，引进大企业、大集团推动重点项目建设。全市力争落地招商引资项目65个，实际到位资金60亿元以上。要引导督促金融部门创新工作思路，加大对重点工程、重点项目的支持力度。切实用好政府投资，重点放在抓配套、搞引导上，并向民生、“三农”、节能环保、技术改造倾斜，发挥好导向作用。

　　各位代表，重点工程建设，事关当前，利在长远。我们所铺开的这些重点工程，绝大部分都是朔州经济社会发展的奠基工程、起跳工程、腾飞工程，将对朔州未来发展起到决定性的作用。今年的重点工程建设，无论项目数量，还是投资规模，都超过以往任何一年，是真正的高潮年。我们一定会以对朔州的明天高度负责的精神，切实抓住大好机遇，身先士卒，靠前服务，科学调度，协调配合，确保工程建设顺利进行，努力为朔州未来发展奠定一个更加坚实的基础！我们一定会以对党对人民高度负责的精神，一丝不苟，严格把关，精益求精抓质量，确保每一项重点工程都建设成为精品工程、德政工程，不负人民期望，经得起历史检验！

　　（三）扎实抓好“三项建设”，全面提升农业农村经济发展水平

　　要以改善农业生产条件、夯实农民增收基础为目标，进一步强化强农惠农政策，突出抓好设施农业、畜牧园区和植树造林三项建设，创新机制，打造精品，示范带动，加快现代农业发展步伐，优化农业农村经济结构，全面提高农业农村经济发展水平。

　　抓好以温室大棚为主的设施农业建设，推动种植业发展上水平。把设施农业作为发展高效优质农业的主要抓手，加大投入，强力推进，不断夯实农业基础，增强农业抵御自然灾害的能力。要按照区域化布局、规模化生产、标准化管理的思路，在各县区选择一些重点乡村进行集中连片建设。每新建一个大棚，市财政将给予2000元补贴。全市要新增各类设施农业3万亩，使设施农业总面积达到4.3万亩，起好示范带头作用，带动全市农业向现代化方向迈进。

　　抓好畜牧园区建设，推动畜牧基地建设上水平。继续加强畜牧基础设施和防疫体系建设。重点扶持奶牛和肉羊两大优势产业，新建奶牛养殖小区50个、肉羊养殖小区30个、青贮窖1500个。争取用三年时间，使90%以上的奶牛进入园区。所有小区都要配套建设饲料加工、畜禽防疫、良种繁育、排污等相关设施。全面实施畜产品安全工程，加强鲜奶收购和饲料、兽药质量安全监管，实现更高水平的规模健康养殖。

　　大规模推进植树造林，推动生态建设上水平。要把生态建设作为朔州最响亮的品牌来精心打造，坚持生态效益、社会效益和经济效益统筹兼顾，山上治本、身边增绿和生态富民同步推进，大力实施造林绿化工程。积极推进集体林权制度改革，调动群众营林造林积极性。建立健全林业建设长效机制，完善“以煤补林”制度，强化生态补偿，逐步提高煤炭可持续发展基金和矿山环境恢复治理保证金用于造林绿化的比例。加大生态建设投入力度，探索建立林业发展基金，实行造林奖励制度。大力推进煤矿生态环境修复治理工程，实施好碳汇造林项目。全市要完成造林33万亩，重点抓好十处万亩以上集中成片绿化工程，并从今年起，用三至五年时间，把怀仁、山阴、朔城区三县区西山生态治理工程连接起来，形成纵贯135公里的京津风沙源治理生态屏障。我们一定要把绿化朔州大地、建设美好家园作为一项崇高的事业和追求。我们坚信，只要我们坚持不懈地抓下去，朔州的山山峁峁终将会变成一片绿色的海洋，朔州大地一定会江山如画，更加靓丽！

　　进一步推进农业产业化发展，提高农业现代化水平。积极支持龙头企业发展，帮助企业解决发展中遇到困难和问题。鼓励龙头企业发展“订单农业”，密切与农民的合作关系，巩固发展“公司+基地+农户”的产业经营模式。鼓励农民成立各类专业合作组织，提高农民组织化程度，增强农业适应市场的能力。健全农业社会化服务体系，完善粮食物流体系，为现代农业发展提供有力支撑。

　　（四）建设全国城乡一体化发展先行区，促进城乡经济社会整体腾飞

　　今年，我市已被国家初步确定为全国城乡经济社会一体化制度建设试验区。这是全国为数不多、我省唯一的此类型国家级试验区。这是我们从根本上打破城乡二元结构，促进城乡互补，实现以工促农、以城带乡、整体腾飞、率先发展的重大战略机遇。我们一定要切实把握好这一重大机遇，将其作为我市扩大改革开放、加快科学发展、加速“两宜”城市建设的重大战略举措来抓，精心组织，扎实推进，努力闯出一条欠发达地区加快发展的新路子。

　　城乡一体化试验区的核心内容，就是要以城乡空间布局、产业发展、基础设施和公共服务、就业和社会保障、社会管理一体化为核心内容，着力推进基础设施向农村延伸、公共服务向农村覆盖，促进土地向规模经营集中、项目向园区集中、农民向二三产业集中、农业人口向城镇转移，构建新型工农关系和城乡关系，努力把我市打造成为全国城乡一体化发展的先行区。近期主要目标就是力争到2015年，全市形成城乡空间形成布局科学合理、功能配套完善、居住相对集中的“1555”发展格局，即容纳50万人的中心城市一座，容纳10万人的卫星城市5座，容纳5000人的小城镇50个，容纳500人以上的中心村落500个；全市农业规模经营面积达到40%，农村劳动力非农就业率达到60%，城市现代化、农村城镇化和城乡一体化的形态初步形成，发展水平跻身全国同类地区前列。要从三个层面同步推进城乡一体化进程。

　　继续加快中心城市建设，高举城乡一体化发展的龙头。要按照“拓展新市区、改造旧城区、提升中心区”的思路，全面加快城市建设，促进城市均衡协调发展，提高城市承载力，增强辐射带动力。“拓展新市区”的重点是，城北盘活长宁街两侧土地，城南架起恢河大桥，西延市府街至西环路，东拓振武街至开发区。“改造旧城区”的重点是，加快推进朔州古城改造，使古城成为城市形象的新亮点；大力推进城中村改造，实现城市协调发展。“提升中心区”的重点是，在安泰街和鄯阳街之间的建成区范围内，精心打造张辽路、开发北路、市府街、安泰街和七里河景观带“两路两街一带”，巩固提升“三化”成果，进一步提升城市品位。今年的主要任务是，继续抓好民福西街立交桥、市府街改造、城市西区水厂等续建工程；新建恢河大桥、交通物流中心、供销社“三农”服务中心、煤炭进出口交易中心、环境监察大楼等重点工程。同时，要围绕创建国家园林城市目标，大力实施绿化工程，全面推进道路绿化、公园绿化、庭院绿化和环城绿化，今年重点搞好张辽路、民福东街高速路出入口、安泰街等重要地段的绿化工程。

　　大力推进特色城镇建设，强化城乡一体化发展的中坚和纽带。各县区要以加快县域经济发展为目标，迅速提升县城的辐射带动功能，强化中心城镇引领吸纳功能，尽快形成一批经济实力较为雄厚、基础设施比较完善的小城镇。要把小城镇作为城乡一体化发展试验区建设的主要着力点，今年争取推开十个试验项目：一是在市区、五个县城和金沙滩镇、北周庄镇、南河种镇实施户籍制度改革，使在城镇稳定就业和居住的农民有序转变为城镇居民。二是在朔城区的北城、南城、神电、北旺庄四个办事处实行户口统一管理。三是在金沙滩镇、北周庄镇和南河种镇三个镇进行强镇扩权试点，每个县选择10个村推行社区化管理模式。四是对三个强镇扩权试点单位和朔城区的四个街道办事处行政区域内的农户宅基地进行确权颁证工作。五是逐步提高标准，推进新农合、新农保与城市医疗保险和低保有效衔接。六是各县区根据不同人群设计不同类型的农村土地承包经营权流转政策措施，促进农村土地向大户集中；七是在平鲁区开展十二年义务教育试点工作。八是在每个县区成立一家村镇银行，合理设置小额贷款公司。九是每县区选择2-3个乡镇以农业生产设施、畜禽、农业重大自然灾害为重点，推行农业政策性保险，建立健全农业生产风险补偿机制。十是建立城乡一体化发展基金。

　　扎实推进新农村建设，夯实城乡一体化的基础。继续推进建制村通水泥路、中小学校舍安全改造、村通广播电视和农村安全饮水工程，改造提升村卫生室，确保年内高标准高质量完成“五个全覆盖”任务。新增新农村建设重点推进村100个，总数达到520个，接近全市行政村总数的三分之一。进一步支持农村特色产业发展，推进“一村一品、一乡一业”工程，增强农村经济实力。组织实施好家电下乡、农机下乡、汽车摩托下乡，抓好“万村千乡市场工程”、“新网工程”、“放心粮油工程”，促进农民消费，活跃农村市场。搞好移民搬迁、整体推进、产业开发、劳动力转移和教育培训，加快农村扶贫开发。继续实施农村困难群众住房解困工程，帮助农民改善居住条件。

　　（五）加快发展各项社会事业，加强社会管理，促进社会和谐稳定

　　优先发展教育。健全教育投入保障机制，加快发展义务教育，促进教育均衡发展。今年重点是大力推进市区范围中小学布局调整和学校建设，切实解决市区上学难、班容量大和学校布局不均衡的突出问题。要利用现师范校舍办好市一中高中部，在开发区、开发北延路经济适用房小区、民福立交桥南和小村拆迁改造区四个地方各规划新建一所幼儿园、小学和初中，并扩建市二中，整合神头电厂区域四所中小学为两所学校。大力发展职业教育。搬迁新建朔州技校（市职业高中），凡没有职业高中或职教中心的县区，要新建一所标准高、功能全的职业中学和职教中心。加快高等教育发展步伐。朔州师专要抓紧配套完善，争取秋季投入使用。能源学院建设年内要取得较大进展。教育是百年大计，孩子是所有父母的希望。我们一定要始终把教育摆在优先发展的地位，不论困难有多大，都要咬紧牙关，舍得投入，着力改善我们的办学条件，提升我们的教育水平，力求让每一个孩子都有一个较高起点的人生起跑线！

　　加快发展医疗卫生事业。深化医药卫生体制改革，努力解决群众看病难、看病贵问题。继续扩大城乡基本医疗保障覆盖面，提高城镇居民基本医疗和新农合的财政补助标准。在60％政府举办的基层医疗卫生机构实施基本药物制度，零差率销售基本药物。加强县乡村医疗机构建设，加快推进达标进程。加强食品药品监督管理，保障群众饮食和用药安全。加强重大传染病防控工作，提高突发公共卫生事件应急处置能力。高度重视人口和计划生育工作，实施部门齐抓共管，强化现代服务手段，确保完成省下达的各项目标任务。

　　进一步稳定和扩大就业。继续实施积极的就业政策，建立健全公共投资带动就业的机制。加强政策支持和就业指导，鼓励高校毕业生到基层、中小企业、非公有制企业就业；鼓励自主创业、自谋职业，以创业带动就业。积极开发公益性岗位，帮助零就业家庭、低保对象、残疾人、破产企业失业职工等困难群体实现就业。加强职业技能培训，提高农民工和城乡新增劳动力的就业能力。维护劳动者合法权益，构建和谐的劳动关系。

　　完善社会保障体系。增加社会保障投入，加大对低收入群体的帮扶救助力度，提高城乡低保标准，提高企业退休人员基本养老金待遇。大力推进各项社会保险市级统筹工作，逐步向省级统筹迈进。积极稳妥地推进事业单位养老保险制度改革。全面实施被征地农民社会保障制度。大力发展社会福利事业和慈善事业，开工建设市社会福利院，完善市流浪儿童救助保护中心，完成复转军人精神病院后续建设工程。实施乡镇敬老院建设工程，力争三年内使农村五保户集中供养率提高到55%。

　　继续推进保障性住房建设。新建廉租住房12.48万平方米、2496套，经济适用房12万平方米、1726套，城市棚户区改造112万平方米、12452套，国有工矿棚户区改造40万平方米、4455套，总计新建保障性住房176.48万平方米、21129套，总投资26.23亿元。增加中低价位、中小户型普通商品房供应，支持居民自主性住房消费。整顿规范房地产市场秩序，促进房地产业平稳健康发展。

　　 大力推进科技创新。要立足产业基础和资源条件，在新能源、新材料、高效节能、煤炭清洁生产和安全生产、特色优质农产品、生态环保等重要领域，组织实施科技攻关。加大高技术成果引进推广力度，应用高新技术改造提升传统产业。加强科技创新体系建设，对企业研发、利用高新技术予以资助。进一步完善农业科技服务网络，加强农业技术推广站建设，积极开展科技下乡活动。加大科技创新投入，形成政府、企业、社会多元投入格局，各级财政科研经费要占到财政支出预算的1%以上。

　　切实加强文化建设。政府要更好地履行发展公益性文化事业的责任，促进社会主义精神文明建设。要加强文化基础设施建设和公共文化资源配置，继续实施乡镇综合文化站建设工程、全国文化资源共享工程，做好博物馆、图书馆、文化艺术中心后续工作。加大文物保护力度，特别要做好基本建设中的文物勘探工作。大力弘扬地方优秀传统文化，积极开展群众性文化体育活动，丰富人民群众精神文化生活。大力弘扬以改革创新为核心的时代精神、以爱国主义为核心的民族精神和朔州精神、右玉精神，激励全市人民始终保持昂扬向上的精神风貌，使朔州大地充满生机与活力。

　　持之以恒地抓好安全生产。牢固树立安全发展理念，毫不松懈地抓好安全生产，推动全市安全生产形势由明显好转向稳定好转转变。进一步强化企业安全生产主体责任。在重点行业推行法定代表人安全生产承诺制。各煤矿兼并重组主体企业要切实负起责任，为每座煤矿配齐配强“六大员”和安全生产管理人员，确保各个关键环节都有人抓、有人管。认真开展安全标准化建设，完成213家企业的标准化建设任务。进一步强化政府安全监管主体责任。全面推行安全监管包保责任制，继续深入开展“安全生产年”活动，强化安全生产专项整治，不断夯实安全生产基础。

　　全面加强社会管理。建立健全重大社会决策、重大工程社会稳定风险评估机制，健全群众权益维护机制。认真做好信访工作，正确处理各类社会矛盾，坚决纠正损害群众利益的突出问题。加强应急管理，完善突发事件应急处置机制。加强社会治安综合治理，严厉打击各类刑事犯罪活动。发展人民防空事业，支持国防后备力量建设，做好双拥工作。加强档案馆库建设和信息化建设，加快达标进程。突出社区建设，年内基本完成市区和各县城社区规划建设任务，做到机构人员、工作场所、经费投入全部落实，并协调管理好物业、治安保卫、医疗、计划生育、环境保护以及流动人口等各项工作，为广大居民创造一个良好的生活环境。

　　各位代表，保障和改善民生，确保社会和谐稳定，是政府义不容辞的责任。我们要尽最大的努力，投入更多的财力和精力，大力推进社会事业发展，多办实事，多做雪中送炭的工作，不断解决群众关注的热点和难点问题，让广大人民群众享受到更多的改革发展成果，生活得更加幸福、更有尊严，让社会更加公正、更加和谐！

　　（六）加强作风建设，加快职能转变，努力建设人民满意的服务型政府

　　新的一年，战胜各种困难和挑战，实现率先发展，政府责任更加重大。我们一定要坚持执政为民的理念，以改革创新的精神，全面履行职能，全面落实责任，不断提高行政能力和行政水平，尽心竭力，恪尽职守，为老百姓服务，为经济社会发展服务，努力建设人民满意的政府。

　　加快职能转变，更加重视公共服务和社会管理。以政府机构改革和事业单位分类改革为契机，切实转变政府职能，既要加强对经济运行的监测和分析，提高经济调节和市场监管的能力；更要增强基本公共服务能力，扩大公共服务范围，提高社会管理和公共服务水平，为各类市场主体创造良好的发展环境，为人民群众提供良好的公共服务。要进一步清理规范行政审批事项，减少审批环节，缩短审批时限，提高行政效率。进一步健全部门协调配合机制，形成强大的工作合力。

　　坚持依法行政，提高政府的公信力和执行力。各级政府要自觉接受人大及其常委会的监督，主动加强与政协的联系，及时办理各类建议和提案。要坚持科学决策、民主决策，不断提高领导科学发展的能力和水平。要坚持依法行政，依法办事，不断加强政府法制建设，加大规范性文件审查力度，强化行政执法监督，全面推进行政复议联办制度，确保规范执法、公正执法、文明执法，坚决杜绝不作为、乱作为现象，严厉查处失职渎职行为。深入推进政务公开，完善各类公开办事制度，保障群众的知情权、参与权、表达权和监督权。要强化政府督查工作，落实行政问责制，确保各项工作落到实处。

　　加强作风建设，树立亲民务实形象。政府工作人员要始终保持良好的精神状态，对群众有感情，对工作有热情，对事业有激情，以高度的责任感和使命感，扎实做好各项工作。坚持一切为了群众，把实现好、发展好、维护好广大人民群众的根本利益，作为工作的出发点和落脚点。坚持一切从实际出发，树立正确的政绩观，求真务实，真抓实干，创造出经得起群众和历史检验的实实在在的政绩。

　　加强廉政建设，让权力在阳光下运行。各级政府要严格执行党风廉政建设责任制，扎实推进惩治和预防腐败体系建设。要坚持勤俭行政，反对铺张浪费，不断降低行政成本。加大行政监察工作力度，加强对重点领域、重点部门、重点项目和关键环节的监督，切实纠正损害群众利益的突出问题和不正之风。继续推进煤焦领域反腐败专项斗争，促进煤炭经济秩序和煤矿安全生产形势持续好转。建立健全群众监督机制，充分发挥新闻舆论的监督作用，创造条件让人民批评政府、监督政府。

　　各位代表，艰辛成就伟业，奋斗创造辉煌。新的形势催人奋进，新的任务艰巨而光荣。让我们在中共朔州市委的坚强领导下，凝聚起全市人民的智慧和力量，再接再厉，开拓进取，全面完成今年经济社会发展和“十一五”规划的各项任务，努力推动朔州在新一轮发展中率先科学发展，在建设塞外最宜居最宜发展城市的伟大征程中不断夺取新胜利！
